# High Resolution Intravital Imaging of Subcellular Structures of Mouse Abdominal Organs Using a Microstage Device

**DOI:** 10.1371/journal.pone.0033876

**Published:** 2012-03-27

**Authors:** Liqin Cao, Satoru Kobayakawa, Atsushi Yoshiki, Kuniya Abe

**Affiliations:** 1 Technology and Development Team for Mammalian Cellular Dynamics, RIKEN BioResource Center, Tsukuba, Ibaraki, Japan; 2 Japan Biological Informatics Consortium, Koto-ku, Tokyo, Japan; 3 Experimental Animal Division, RIKEN BioResource Center, Tsukuba, Ibaraki, Japan; The Beatson Institute for Cancer Research, United Kingdom

## Abstract

Intravital imaging of brain and bone marrow cells in the skull with subcellular resolution has revolutionized neurobiology, immunology and hematology. However, the application of this powerful technology in studies of abdominal organs has long been impeded by organ motion caused by breathing and heartbeat. Here we describe for the first time a simple device designated ‘microstage’ that effectively reduces organ motions without causing tissue lesions. Combining this microstage device with an upright intravital laser scanning microscope equipped with a unique stick-type objective lens, the system enables subcellular-level imaging of abdominal organs in live mice. We demonstrate that this technique allows for the quantitative analysis of subcellular structures and gene expressions in cells, the tracking of intracellular processes in real-time as well as three-dimensional image construction in the pancreas and liver of the live mouse. As the aforementioned analyses based on subcellular imaging could be extended to other intraperitoneal organs, the technique should offer great potential for investigation of physiological and disease-specific events of abdominal organs. The microstage approach adds an exciting new technique to the *in vivo* imaging toolbox.

## Introduction

Intravital microscopy has been rapidly gaining prominence and increased usage in biomedical research in recent years [1], [2], [3], [4], [5], [6]. The advantages of these *in vivo* imaging techniques are that they allow observations and measurements of cell behaviors and complex biological interactions in their natural state, which is crucial to understand fully the underlying biology. Some intravital imaging techniques allow for repeated imaging of the same individual. This potential for longitudinal study enables the researcher to identify inter-animal variability in physiological response to changing environments in a manner that is not possible to achieve by other means and to reduce the number of animals required for a given study.

*In vivo* imaging of tissue architecture and single cells in live animals has addressed a number of central questions in biology [1], [2], [3], and imaging at a subcellular level has further opened up a new era for investigating dynamics of intracellular events [4], [7], [8]. For instance, *in vivo* imaging of the live mouse brain and calvarial bone marrow with subcellular resolution has revealed unexpected details of the dynamic nature of synaptic structures through development, reshaped the understanding of neurodegenerative diseases and of immune surveillance function of microglia and corroborated an hypothesis of platelet generation [9], [10], [11], [12]. However, imaging intact abdominal organs in live mice has long been a challenge because of tissue motion caused by respiration and heartbeat. In anesthetized mice, motions in the liver arising from breathing or heartbeat occurred once every 1–2 sec with an amplitude of approximately 1200 µm and 10 times per sec with an amplitude of approximately 10 µm, respectively [13]. Such motion limited imaging studies of intraperitoneal organs at cellular resolution except for a few reports on the kidney and small intestine at a subcellular level [14], [15], [16], [17], [18], [19], [20], [21]. In those kidney studies, the left kidney was imaged using an inverted microscope, taking advantage of the longer left renal pedicle to facilitate kidney exteriorization and limit its movement [15], while in the small intestine studies a segment of small intestine was exteriorized for imaging [19], [20], [21]. Recently, one group established a stabilizing system to reduce organ motion artifacts by applying pressure on the organ's surface [22]. The system showed success in improving deep tissue imaging of the intestine; however, the imaging of various abdominal organs was still confined to a cellular level of resolution [22]. In addition, the pressure applied might cause unwanted deleterious effects on the organs. Thus, it is of great importance and urgency to develop techniques that help to minimize tissue motion and facilitate imaging of abdominal organs at subcellular level *in vivo* to enable kinetic analyses of intracellular processes in those organs. Furthermore, such techniques would offer the opportunities to observe primary tumors of internal organs orthotopically and should provide unique and novel insights into the growth, intracellular biological processes and therapeutic response of these tumor cells in their native environments, given that ectopic models are mainly used for current microscopic imaging studies of abdominal organ tumors in mice [23].

In this study, we designed a microstage device that can effectively reduce the motion of mouse abdominal organs without producing organ lesions. Using this device in conjunction with an upright microscope equipped with a very thin, stick-type objective lens [14], high-resolution images of abdominal organs can be acquired. We present here the quantitative comparison of gene expressions and subcellular structures in cells under different physiological conditions, and the real-time visualization of cellular processes and organelle dynamics in some intraperitoneal organs of live mice, of which the abovementioned quantitative analyses and time-lapse tracking of subcellular organelles in the pancreas and liver have not to our knowledge been reported before.

## Results and Discussion

### Design of the microstage device for intravital imaging of abdominal organs

**Figure 1:**
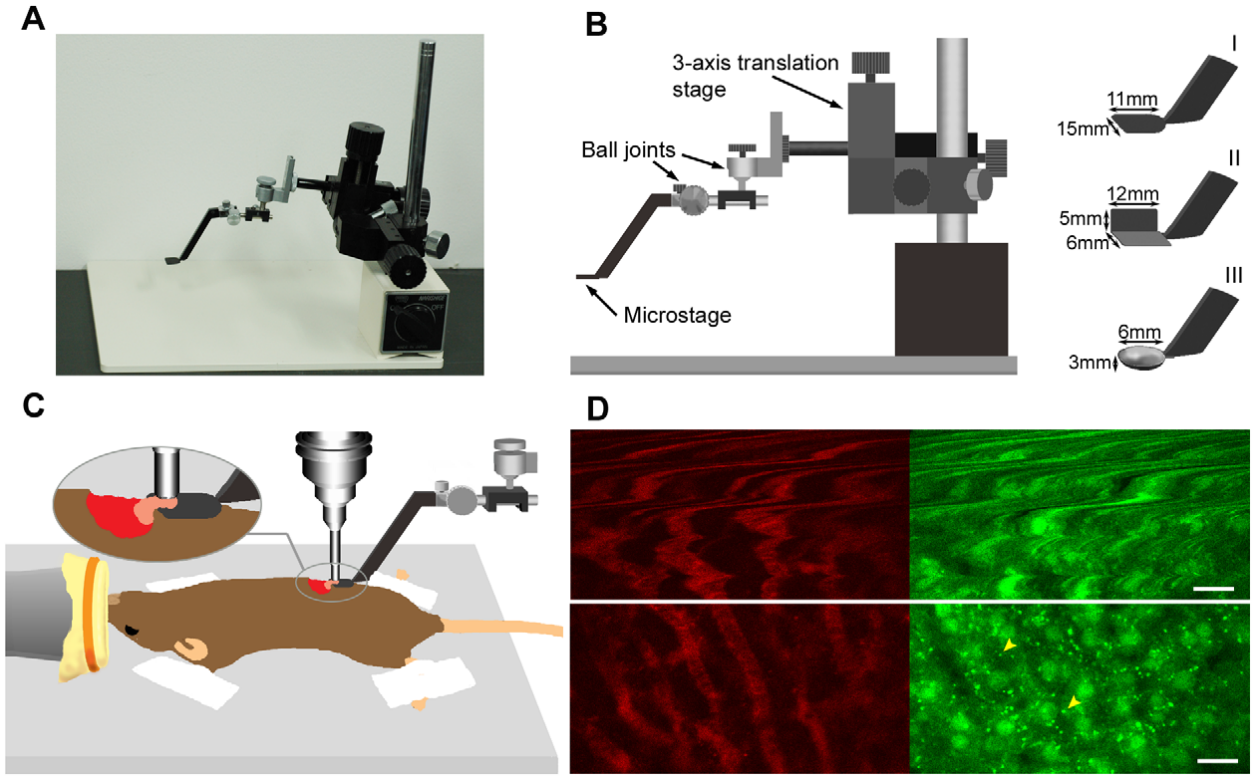
Effect of the microstage device on image stability and quality. (A) Microstage device. (B) Illustration of the microstage device and microstages of three types. (C) Illustration showing that a portion of the pancreas of an anesthetized mouse was positioned on the microstage and imaged using a stick-type water immersion lens. (D) *In vivo* images of liver vasculature (left, red, labeled with AngioSense 750 IVM) and liver cells (right, green) of the GFP–LC3 mouse taken without (top row) and with (bottom row) the microstage device. Arrows depict GFP-labeled autophagic membranes. Individual images in D are 512×256 pixel areas cropped from 512×512 pixels. Scale bars, 20 µm.

Our microstage device mainly consists of a custom-made metal microstage, a 3-axis (*x*, *y* and *z*) translation stage and two ball joints (Fig. 1A, B and Figure S1A, B). The microstage provides a solid support platform for organ tissues. For imaging, the stage is raised slightly so that the tissue portion on the stage is relieved of direct contact with the main body of the mouse and a small portion of the tissue forms a bridge between the organ on the stage and the body, which dampens the tissue motion to a great extent (Fig. 1C). The translation stage and ball joints enable the microstage to move in three dimensions (*x*, *y* and *z*) and to rotate and tilt about the *x*, *y* and *z* axes freely. Hence, the microstage can easily gain access to various intraperitoneal organs such as the liver, kidney, pancreas and spleen. We designed three types of microstages to achieve optimal tissue stabilization for organs of different morphology, specifically, a flat, spade-like shape for the liver, pancreas and spleen that have a relatively even dorsal surface, a plate folded at a 90° angle for the kidney that has a three-dimensional (3D) curved surface and a spoon-shape type for the ovary and testis that are nearly spherical in shape (Fig. 1B and Figure S1C–E).

To evaluate the capability of the microstage device to stabilize motion, we intravenously injected the fluorescent intravascular probe, AngioSense 680 IVM or AngioSense 750 IVM, into GFP–LC3 transgenic mice in which autophagic membranes (including phagophores and autophagosomes) were labeled by GFP as punctate dots [24], and then imaged pancreatic and liver tissues with and without a microstage using the same image acquisition parameters (scanning frequency, resolution, laser power) in the same animals. We chose the liver and pancreas for this testing as these organs would be most affected by motions among all abdominal organs owing to their anatomical location near the thoracic cavity. Comparisons of the images demonstrated that the use of the microstage effectively reduced tissue motions and significantly improved the image integrity and resolution (Fig. 1D and Movies S1 and S2). In images obtained without a microstage, cell morphology and vascular structures were severely distorted by motion and subcellular structures such as GFP-marked autophagic membrane dots (∼1 µm in diameter) were unresolved (Movies S1, top and S2, top). In sharp contrast, in images attained with a microstage, motion artifacts were greatly suppressed in the tested organs (Movies S1, bottom and S2, bottom). Motion reduction by the microstage appeared to be more robust for the pancreas than the liver, probably because of the softer nature of pancreas. Nevertheless, in high-resolution images of the liver, GFP-positive autophagic membrane dots were clearly resolved in the cells, and moving blood cells were evident as dark foci in the vasculature (Fig. 1D, bottom and Movie S2, bottom). As shown in Movie S1, images of the pancreas were greatly improved by the use of the microstage. Image distortions are mainly caused by respiration and heartbeat occurring once every 1–2 sec and 10 times per sec, respectively [13]. The video was recorded for 6 sec, clearly revealing that pancreatic tissue motions caused by both breathing and heartbeat had been almost completely suppressed by the microstage. For the liver, the video was recorded for 53 sec. While streak artifacts occurred in some images as a result of the breathing movements, the high resolution images of the GFP-dots were acquired when the microstage was applied, demonstrating that the motion artifacts caused by heartbeat were effectively suppressed and the image distortions due to respiration were also reduced by the microstage (Movie S2). Therefore, we would be able to perform quantitative image analysis of subcellular structures stabilized by the microstage using the movie data made by combining images with no apparent image artifacts extracted from the raw dataset (Movie S3). The movie could be further improved by subsequently registering the image series using the StackReg plugin of Image J software if necessary (Movie S4). Since the pancreas and liver would be most affected by motions among all abdominal organs as described, the results shown here suggest that the microstage device would be a very effective tool to reduce tissue motions and enable subcellular intravital imaging for virtually all abdominal organs.

We next tested the applicability of the system for long-term imaging by performing extended time-lapse imaging of the pancreas (Movie S5, 12.5-min total duration) and liver (Movie S6, 120-min total duration) in GFP–LC3 mice. Movies S5 and S6 clearly show that serial images with subcellular resolution were able to be collected over periods ranging from minutes to hours for these two tissues. The stick objective used in this study had a limited field of view (220 µm), posing difficulty for relocating the same imaging area in multiple imaging sessions. However, the system is also compatible with conventional microscope objectives providing broader fields of view (from 300 µm to 3000 µm, personal communication). Using wide field-of-view images of tissue architecture taken with the conventional objectives as landmarks we were able to re-image the same area at subsequent time points (data not shown). Taken together, the microstage technique allowed high-resolution repetitive long-term imaging of intraperitoneal organs in live mice, and should facilitate studies of important biological questions in these organs.

### Quantitative analysis of subcellular structure dynamics and gene expressions

**Figure 2:**
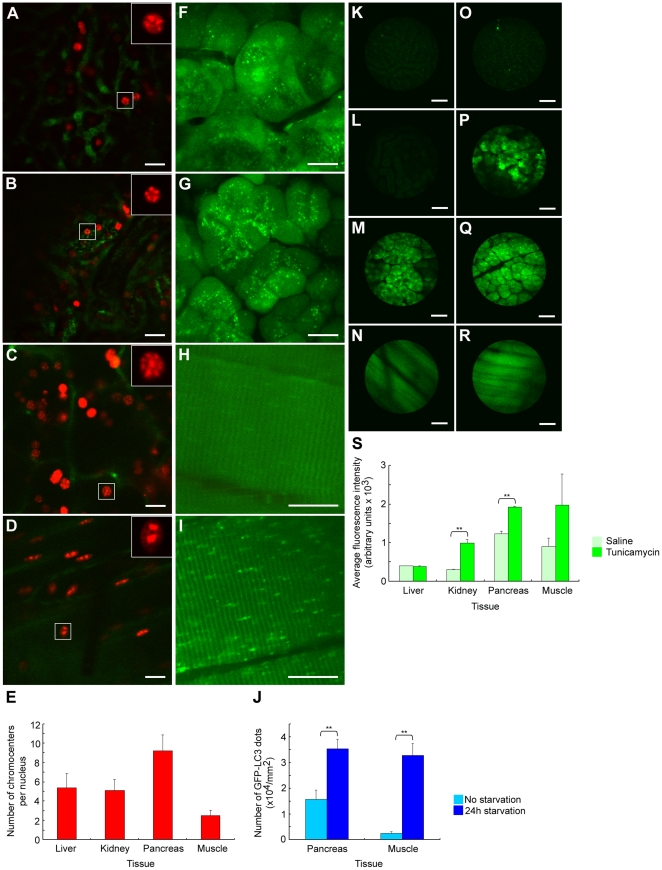
*In vivo* imaging of subcellular structures and gene expression in mouse abdominal organs with the microstage device. (A–D) Highly methylated chromocenters labeled by mRFP–MBD-nls in cells of liver (A), kidney (B), pancreas (C) and skeletal muscle (D) of mRFP–MBD-nls transgenic mice. Green pseudocolor shows the vasculatures labeled with an intravenous injection of AngioSense 750 IVM. Insets: magnified pictures of the boxed area in corresponding images. (E) Quantitative analysis of chromocenter numbers in cell nuclei of four types of tissues. Counts of 24, 23, 23 and 32 cell nuclei were analyzed for the liver, kidney, pancreas and skeletal muscle, respectively. (F–I) Autophagy in pancreatic acinar cells (F, G) and skeletal muscle (H, J) of GFP–LC3 mice before (F, H) and after (G, I) 24 h starvation. (J) Quantitative analysis of autophagy in response to 24 h starvation. The numbers of GFP–LC3 dots were counted and divided by the corresponding area (n = 5 image fields). (K–R) Fluorescence intensity in liver (K, O), kidney (L, P), pancreas (M, Q) and skeletal muscle (N, R) of ERAI-transgenic mice injected with saline (K–N) or tunicamycin (O–R). (S) Quantitative analysis of fluorescence intensity in tissues under different physiological conditions (n = 3 image fields). Results in E, J and S represent the mean ± SD. **P<0.01. Images in A–D and F–I are 512×512 pixels. Images in K–R are 320×320 pixels. Scale bars, 20 µm (A–D, F–I) and 50 µm (K–R).

Using the microstage device, we first imaged and quantified subcellular structures and gene expressions in the abdominal organs and the skeletal muscle of three transgenic mouse strains. The skeletal muscle served as a control since the skeletal muscle can be immobilized easily (see Materials and Methods). The results showed that the quality of abdominal organ images acquired with the microstage were comparable to that of the skeletal muscle (Figure 2).

Our previous study demonstrated that the EGFP-MBD-nls fusion gene product could faithfully trace global DNA methylation changes in living mouse embryonic stem cells during cell differentiation [25]. We established a transgenic mouse line, in which mRFP–MBD-nls fusion protein is ubiquitously expressed and used them for intravital imaging. Figure 2A–D shows subnuclear structures, highly methylated centromeric heterochromatin aggregates (chromocenters), which were intensely labeled with the mRFP–MBD-nls fusion protein in the nuclei of four types of tissues of the live mRFP–MBD-nls mice. Interestingly, the number of the chromocenters per nucleus differed among cell types. For example, two large chromocenters could be almost always found in skeletal muscle cells. As shown in Figure 2E, chromocenter numbers varied from 2.5±0.6 (n = 32) in skeletal muscle cells to 9.2±1.7 (mean ± SD, n = 23) in pancreatic acinar cells, and similar numbers of chromocenters were reproducibly found in cells of the same type. Thus the number of methylated chromocenters could be determined under strict developmental control. In Figure 2A–D, some out-of-focal-plane blurry nuclei were observed because the IV100 microscope is not a true confocal system and provides only a degree of 3D optical sectioning that helps to reduce but not completely exclude out of focus light [13]. Further we noticed that the proportion of nuclei with unresolved subnuclear structures was decreased in the image when the focal plane was adjusted toward deeper regions in the tissue (Figure S2), and reasoned that more nuclei in superficial regions of the tissue tended to be slightly out of focus comparing to those in deeper regions because of the uneven nature of the organ surface. The observation of chromocenters at high resolution in intact abdominal organs should enhance the *in vivo* investigation of how DNA methylation is regulated under various physiological conditions and might provide new insights into the epigenetic etiology of diseases.

Figure 2F–I shows starvation-induced autophagic responses in pancreatic acinar cells and skeletal muscle cells of the GFP–LC3 mice. The GFP–LC3 reporter specifically labels autophagic membranes as bright punctate structures and is an ideal marker for examining autophagic activity in organs [24]. Before the starvation treatment, acinar cells appeared to possess many more GFP–LC3-positive dots than did muscle cells (Fig. 2F, H), indicating that the basal level of autophagy in acinar cells was relatively high, which is consistent with the reported results obtained from fixed tissue sections [24]. We quantified the number of GFP–LC3 dots under normal and 24 h starvation conditions in the same three sibling mice and found that this number was significantly increased in both pancreatic acinar cells and muscles after starvation (Fig. 2J). Our quantitative analyses of the formation of GFP–LC3 dots during starvation indicated that the dot numbers estimated from *in vivo* imaging data (Fig. 2J) are similar to those attained by examining the sections of fixed samples [24]. This result demonstrated that the microstage technique facilitated subcellular-resolution longitudinal imaging of mouse abdominal organs.

Figure 2K–R shows endoplasmic reticulum (ER) stress indicated by F-XBP1ΔDBD-Venus reporter gene expression in several organs of ERAI transgenic mice [26]. Iwawaki et al. have reported that tunicamycin treatment induced ER stress in mice, resulting in detectable fluorescence in tissues of ERAI mice but not wild-type mice. Further, the fluorescence intensity correlated with ER stress *in vivo* [26]. In our control experiment when the ERAI mouse received an intraperitoneal injection of saline and was imaged 24 h later, there was little fluorescence in the liver but some basal fluorescence in the pancreas, kidney and skeletal muscle (Fig. 2K–N). When the mouse received an injection of tunicamycin and was imaged 24 h later, *in vivo* imaging demonstrated that the fluorescence intensity showed virtually no change in the liver, a significant increase in the pancreas and kidney and also an increase in the skeletal muscle but not to a significant level (Fig. 2O–S). These results suggest that both basal and tunicamycin-induced ER stresses can vary between organs.

### Real-time observation of cellular processes and organelle kinetics

**Figure 3:**
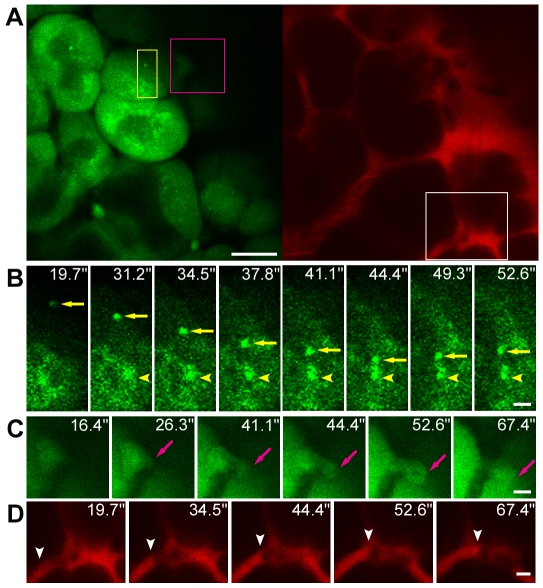
*In vivo* imaging of cellular processes in the pancreas of live GFP–LC3 mice starved for 24 h (see also Movie S5). (A) Pancreatic acinar cells (left) and vasculature (right). (B) Time-lapse images of autophagosome movements in the boxed region (yellow) in A at the indicated times. Two GFP–LC3 dots exhibited distinct behaviors; one showed rapid displacement (yellow arrow), while the other remained stationary (yellow arrowhead). (C) Time-lapse images of an event similar to exocytosis in the boxed region (magenta) in A at the indicated times. Magenta arrows designate the protrusion-like profile. (D) Time-lapse images of pancreatic microcirculation obtained from the boxed region (white) in A at the indicated times. Individual cells appeared as dark foci in the vasculature. White arrowheads mark progress of a single cell. Scale bars, 20 µm (A), 2 µm (B) and 5 µm (C, D).

As a second challenging examination, we studied dynamics of rapidly moving autophagosomes in the pancreas of live GFP–LC3 mice. Both autophagosomes and phagophores were observed as GFP-positive dots [24]. A previous study demonstrated that autophagosomes are dynamic organelles moving along microtubules, whereas phagophores remain immobile within the cell [27]. Therefore, we considered immobile GFP dots as phagophores and used them as reference landmarks for correct estimation of the displacement of autophagosomes during a given time interval (Materials and Methods). In acinar cells, the autophagosomes exhibited either rapid, long-distance directional movements or short movements (Fig. 3B, Movie S7 and S8), while the phagophore remained stationary (yellow arrowhead in Fig. 3B, Movie S7). The maximum speed of autophagosome movement (yellow arrow in Fig. 3B, Movie S7) which we measured *in vivo* was 0.65 µm/s, which is comparable to the result obtained using cultured Chinese hamster ovary cells *in vitro* [27]. Minor shape distortions or blurring streaks were observed in a few frames of Movie S7 and S8, which were image artifacts resulting from tissues motion. While observing autophagosome movements, we occasionally observed events that appeared similar to exocytosis. Figure 3C (also see Movie S7) shows a representative time course of one such event (magenta arrow). Although the possibility of tissue drift in the *z*-axis cannot be completely excluded, it appeared that a cell protrusion-like structure was first formed, and then gradually extended and budded off from the cell, leading to release the cell components into the extracellular space. In addition, the movements of cells in the vasculature could be traced (white arrowhead in Fig. 3D) in the same image field.

### Generation of three-dimensional images

Because organ motions were effectively minimized by the microstage device, it became possible to acquire optical sections in live mouse abdominal organs such as the liver. We acquired *z*-stacks of microvasculature in the liver (79 µm deep sections) and gastrocnemius muscle (103 µm deep) of GFP–LC3 mice and subsequently reconstructed 3D images from these stacks. The liver proved to have larger vessels and higher vessel densities than the gastrocnemius muscle (Movie S9).

In summary, our simple microstage device can effectively reduce abdominal organ motions, which have been a serious obstacle to *in vivo* imaging. In combination with a stick-type lens mounted in a IV100 laser scanning microscope, this system enabled us to investigate subcellular structures and gene expression patterns in cells quantitatively, to monitor organelle dynamics, cellular secretion-like events and blood cell flow in vasculature in real time and to conduct optical sectioning in some abdominal organs, namely the pancreas and liver of live mice. Such analyses could be extended to virtually all other organs within the abdominal cavity as we have demonstrated in the kidney in this study. The microstage device seemed to bring about negligible impairments to organs, as the mice imaged repeatedly with this device had a normal life expectancy and behaved normally. This provided great potential for longitudinal subcellular *in vivo* imaging of the same animal. Here we have used our microstage to facilitate the longitudinal comparison of autophagy in response to starvation in GFP–LC3 mice. Nevertheless, assessments of possible complications that may relate to chronic observation are needed for specific research studies. Moreover, the device can be modified simply and applied to other upright imaging systems including confocal and two-photon microscopes. We believe that our technique opens up a new avenue for studying cellular and molecular functions, subcellular organelle dynamics, disease progression (including orthotopic cancer models) and therapeutic responses at the subcellular level in abdominal organs *in vivo*. It should lead to breakthroughs in understanding biological processes and disease mechanisms in abdominal organs.

## Materials and Methods

All animal experiments were approved by the Institutional Animal Experiment Committee of RIKEN BioResource Center (No. 09-006).

### Intravital laser scanning microscope, microstage device and fluorescent probes

We employed an intravital laser scanning imaging system (IV100, Olympus) in combination with a custom designed microstage device for imaging subcellular structures and organelles in real time in live mice. The IV100 carries a set of lasers whose excitation capabilities span from green to near-infrared (488, 561, 633 and 748 nm) and can be used to image three wavelengths simultaneously. Its stick-type objective lenses facilitate access to virtually any abdominal organ and its tiltable microscope head provides optimal excitation for imaging the uneven and rounded surfaces of abdominal organs. The small diameter of the optical fibers capturing emission together with the relatively high numerical aperture (NA) of the objective lenses provides a degree of optical sectioning without requiring a pinhole [14].

Type I and II microstages were used for imaging different types of organs in this study. These microstages are detachable from the system and can be mounted easily on the device interchangeably during the experiment, allowing imaging of different organs at high resolution in the same animal. A near-infrared fluorescent probe (AngioSense 680 IVM or AngioSense 750 IVM, PerkinElmer) was used to highlight the microvasculature of organs. The probe circulates through blood vessels for extended time (approximate half-life in plasma is 6 h).

### Animal models and *in vivo* imaging procedures

Three transgenic mouse models expressing fluorescent proteins were used. The mRFP–MBD-nls mouse (RBRC01739) carries the mRFP reporter gene fused to methylated DNA binding domains (MBD) and the nuclear localization signal (nls) sequences of human *MBD1*. The transgene is essentially identical to the EGFP-MBD-nls fusion gene that has been published [25] except that the mRFP gene, instead of the EGFP gene, is used in the construct. It is driven by the CAGGS promoter [28] to express mRFP–MBD-nls ubiquitously. This fusion protein binds exclusively to methylated DNA in nuclei, enabling direct visualization of global DNA methylation patterns in individual cells. The GFP–LC3 mouse (RBRC00806) expresses the GFP–LC3 fusion product ubiquitously. In this case, GFP–LC3 specifically labels autophagosomes and autophagic isolation membranes (phagophores, the precursors of autophagosomes), allowing the observation of occurrence of autophagy in various tissues [24]. The ERAI (endoplasmic reticulum stress-activated indicator) mouse (RBRC01099) expresses F-XBP1ΔDBD-Venus fluorescent protein in the cytosol in a variety of organs when ER stress is induced and therefore can be used as a model for monitoring this *in vivo* [26]. All three transgenic mouse models were obtained from the RIKEN BioResource Center (http://www.brc.riken.jp/inf/en/). In all live animal experiments, adult mice were anesthetized with 2% (vol/vol) forane in 2 L/min O_2_ delivered by facemask, and were placed on a heated plate to maintain the body temperature. Following anesthesia, 100 µL aliquots of AngioSense 680 IVM or AngioSense 750 IVM were administrated intravenously immediately prior to imaging. For ERAI mice, saline or 50 µg tunicamycin (Sigma-Aldrich) in saline, which induces ER stress, were injected intraperitoneally 24 h before imaging.

Mice were shaved and swabbed with ethanol at the desired surgical sites before the experimental session. For imaging the liver or pancreas, a type I microstage was used. First, a small incision was made through the abdominal skin to expose the organ of interest. A portion of liver or pancreas was carefully lifted with a blunt forceps and mounted on the microstage. Then the microstage was raised slightly above the mouse main body so that a small region of the tissue formed a bridge connecting the tissue portion on the microstage to that with the main body (Fig. 1C). As the liver and pancreas are soft tissues, this tissue bridge served as a buffer zone to lessen greatly the influence of the cardiac and respiratory motions on the tissue portion on the microstage. For kidney imaging, a small dorsal incision was made. A type II microstage was turned at a suitable angle so that its horizontal plane supported the kidney from the underneath and its vertical panel protected the kidney against the motion impact from the main body laterally. For skeletal muscle imaging, a small incision was performed in the hind limb skin and the gastrocnemius muscle was identified for imaging. Clay was used to fix the limb on the heated plate to reduce motion artifacts. After positioning the specimen under the stick-type objective, the microscope lens was slowly lowered until the specimen was in focus. We kept the organ moist by irrigating the exposed tissue regularly with sterile saline during the imaging session. For repetitive imaging, we sutured the skin over the incision and allowed the mice to recover for at least one week before the second imaging session.

### Imaging parameters and data processing

The 488, 561, 633 and 748 nm lasers were used for imaging GFP, RFP, AngioSense 680 IVM and AngioSense 750 IVM, respectively. All the imaging experiments were performed with a 27×stick-type water-immersion objective lens (0.67 NA, 200 µm working distance).

Images for quantitative analysis were acquired at 0.6, 0.9 or 1.7 Hz frame rates. To compare the pattern of nuclear organization among tissues, the numbers of highly methylated chromocenters labeled by the mRFP–MBD-nls protein were counted in randomly selected single nuclei (n>20) from liver, kidney, pancreas and gastrocnemius muscle of mRFP–MBD-nls mice, respectively. To assess autophagic activity, tissues of the same GFP–LC3 mice were imaged before and after 24 h starvation treatment, and the numbers of GFP–LC3-positive puncta were counted in five independent image fields for individual tissues. To monitor ER stress, the ERAI gene expression levels in ERAI mice were determined by measuring the average fluorescence intensity in single optical sections in various tissues using the IV10-ASW image processing software (Olympus). For each tissue, estimates from three independent sites were averaged and the resulting value represented the ERAI gene expression level of the tissue. Results of quantitative analysis are presented as the mean ± SD. The differences between experimental groups were evaluated using Student's *t* test. A p-value<0.05 was considered significant.

Video data were attained using the GFP–LC3 live mice. Movies S1 and S2 show single *z*-planes of the pancreas and liver scanned continuously at 0.9 Hz frame rate for approximately 6 and 53 s, respectively. The movies were constructed using raw image data that were corrected only for brightness and contrast. Movie S3 was extracted from Movie S2 (bottom row) by removing the frames with streak artifacts from the original stacks. Subsequently, the images of Movie S3 were aligned using the auto-alignment StackReg plugin of Image J software, and the unwanted black edge areas produced by the alignment process were cropped off the stacks. The resulting image series were used to create Movie S4. Movies S5 and S6 show the time-lapse imaging of the pancreas and liver at single focal planes over 12.5 and 120 min, respectively. The image series of the pancreas were collected every 30 s, while those of the liver were collected every 60 s for the first 20 min followed by every 10 min up to a total time of 120 min. All images were acquired at 0.9 Hz frame rate. Focus drift and lateral tissue shift occurred over the course of extended imaging were manually corrected when necessary, by repositioning the objective lens slightly between two image acquisition sessions. Post-acquisition, image stacks were aligned using Image J software after distorted frames caused by tissue motion were subtracted. Unwanted edges were cropped off images, and the resultant stacks were used to generate Movies S5 and S6. Movies S7 and S8 are real-time images of the pancreas acquired at single focal planes at 0.6 Hz frame rate. Images severely distorted by tissue motion artifacts were removed from the image stacks. As a minor lateral *x*–*y* displacement of image inevitably occurred between consecutive frames during *in vivo* imaging, to estimate the movement of autophagosomes in cells, structures staying immobile within cells over time were chosen as a reference. Once the centroid pixel of the reference was determined (Xr, Yr), the displacement (d) of an autophagosome (centroid Xa, Ya) between two time points (0, t) was calculated using the following equation, d^2^ = [(Xat−Xrt)−(Xa0−Xr0)]^2^+[(Yat−Yrt)−(Ya0−Yr0)]^2^, and the speed of the autophagosome movement was estimated as d/t. The raw images of Movie S9 were acquired for the liver and gastrocnemius muscle at 1.7 Hz frame rate over a 79 µm axial range at 1 µm spacing and over a 103 µm axial range at 1 µm spacing, respectively. Distorted images and the images with a considerable lateral displacement compared with that taken at the initial time point were eliminated from the stacks. The 3D videos in Movie S9 were *z*-dimension projections produced using the IV10-ASW software (Olympus). Final image preparation, including adjustment in brightness and contrast, was conducted using IV10-ASW software.

## Supporting Information

Figure S1 **Tissue stabilization for intravital imaging of mouse abdominal organs.** (A, B) Stick-type objective (arrow) and microstage device (arrowhead). (C–E) Type I, type II and type III microstages were used to minimize tissue motion of the liver (C), kidney (D) and testis (E), respectively.
(TIF)

Figure S2 ***In vivo*** **imaging of the pancreas of the mRFP–MBD-nls transgenic mouse.** Highly methylated chromocenters were labeled by mRFP–MBD-nls in pancreatic acinar cells. The axial positions marked in each frame denote the focal plane relative to the starting position (tissue surface). Arrows depict nuclei whose chromocenters became well resolved when the focal plane was adjusted toward deeper regions. Scale bar, 20 µm.
(TIF)

Movie S1 **Comparison of videos of pancreatic tissue from GFP–LC3 mice acquired without (top row) and with (bottom row) the microstage device.** GFP-positive pancreatic acinar cells (left, green). Vasculature (right, red, labeled with AngioSense 750 IVM) in the same image field. Both videos were acquired using the same image acquisition parameters and were constructed without image alignment and *z*-selection of individual planes. Scale bars, 20 µm.
(MOV)

Movie S2 **Liver tissue of GFP–LC3 mouse acquired without (top row) and with (bottom row) the microstage device.** Liver cells (left, green). Vasculature (right, red). Both videos were acquired using the same image acquisition parameters and were constructed without image alignment and *z*-selection of individual planes. Scale bars, 20 µm.
(MOV)

Movie S3 **Videos of the GFP-LC3 mouse liver tissue acquired with the microstage device.** This movie was extracted from Movie S2 (bottom row) by removing the frames with streak artifacts from the image stacks. Liver cells (left, green). Vasculature (right, red). Scale bar, 20 µm.
(MOV)

Movie S4 **Videos of the GFP-LC3 mouse liver tissue.** This movie was constructed using the image series of Movie S3 after having applied the alignment procedure. Liver cells (left, green). Vasculature (right, red). Scale bar, 20 µm.
(MOV)

Movie S5 **Time-lapse imaging of the GFP-LC3 mouse pancreas.** Image series were recorded every 30 s, 12.5-min total duration. Pancreatic acinar cells (left, green). Vasculature (right, red). Scale bar, 20 µm.
(MOV)

Movie S6 **Time-lapse imaging of the GFP-LC3 mouse liver.** Image series were recorded every 60 s for the first 20 min followed by every 10 min up to a 120-min total duration. Liver cells (left, green). Vasculature (right, red). Scale bar, 20 µm.
(MOV)

Movie S7 **Videos of cellular processes in the pancreas shown in** **Figure 3** **.** The yellow box represents the area where two GFP–LC3 positive dots exhibited distinct behaviors in an acinar cell (yellow arrow and yellow arrowhead). The magenta box specifies the location where an event that appeared similar to exocytosis (magenta arrow) took place. The white box shows the region where a cell (white arrowhead) moved in the vasculature. Scale bar, 20 µm.
(MOV)

Movie S8 **Videos of the pancreas of a GFP–LC3 mouse after 24 h starvation.** Pancreatic acinar cells (left, green). Vasculature (right, red). Scale bar, 20 µm.
(MOV)

Movie S9 **Videos of the 3D reconstructions of GFP–LC3 mouse liver (left) and skeletal muscle (right) vasculatures.**
(MOV)
