# Supplementary figures and images for: High Resolution Intravital Imaging of Subcellular Structures of Mouse Abdominal Organs Using a Microstage Device
Source: PLoS One. 2012 Mar 27;7(3):e33876. doi: 10.1371/journal.pone.0033876 (PMC3313950; doi:10.1371/journal.pone.0033876)

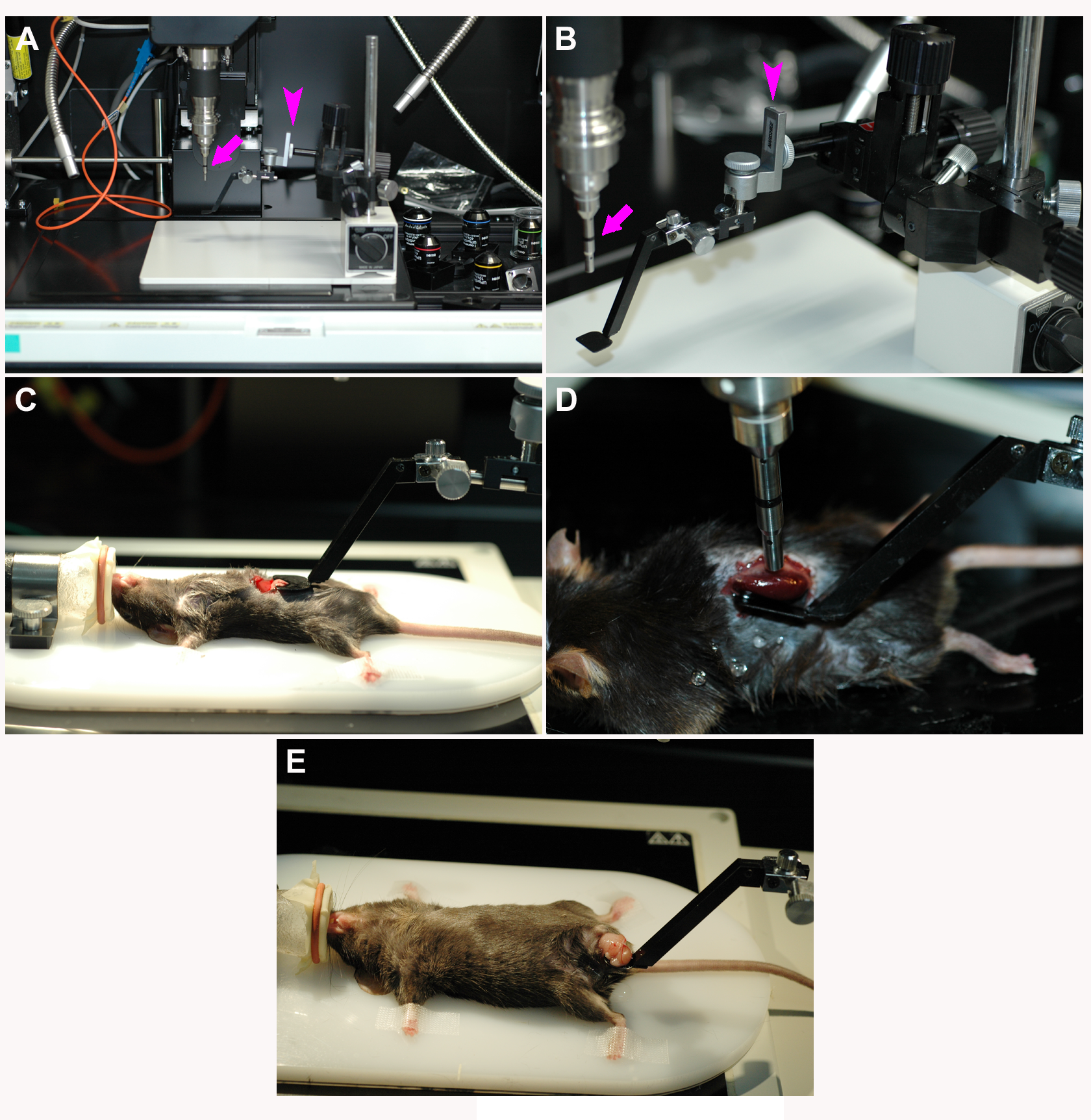

Supplement: Figure S1 — Tissue stabilization for intravital imaging of mouse abdominal organs. (A, B) Stick-type objective (arrow) and microstage device (arrowhead). (C–E) Type I, type II and type III microstages were used to minimize tissue motion of the liver (C), kidney (D) and testis (E), respectively. (TIF) [file pone.0033876.s001.tif]

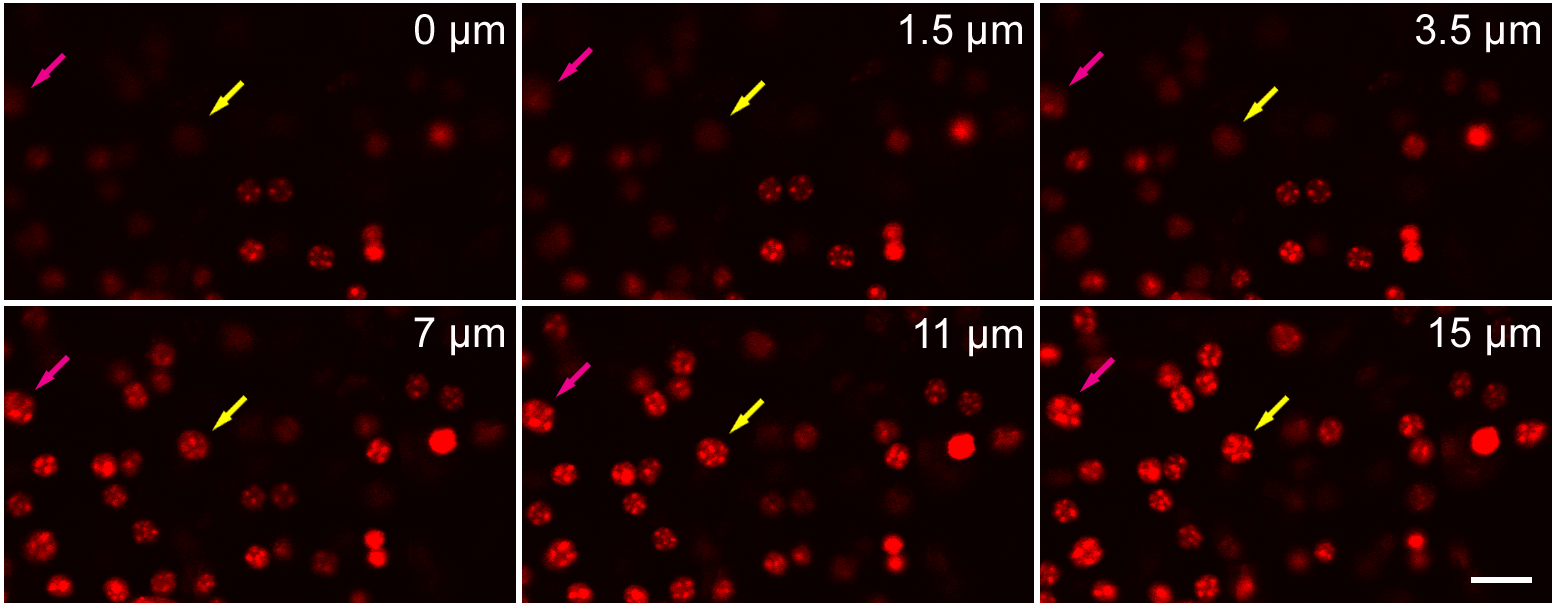

Supplement: Figure S2 — In vivo imaging of the pancreas of the mRFP–MBD-nls transgenic mouse. Highly methylated chromocenters were labeled by mRFP–MBD-nls in pancreatic acinar cells. The axial positions marked in each frame denote the focal plane relative to the starting position (tissue surface). Arrows depict nuclei whose chromocenters became well resolved when the focal plane was adjusted toward deeper regions. Scale bar, 20 µm. (TIF) [file pone.0033876.s002.tif]
